# Predictors for the development of preoperative oxygenation impairment in acute aortic dissection in hypertensive patients

**DOI:** 10.1186/s12872-020-01652-5

**Published:** 2020-08-10

**Authors:** Xuemin Zhao, Mengjun Bie

**Affiliations:** 1 Department of Cardiology, The First Branch Hospital, The First Affiliated Hospital of Chongqing Medical University, Chongqing, 400016 China; 2 Department of Cardiothoracic Surgery, The First Affiliated Hospital of Chongqing Medical University, Chongqing, 400016 China

**Keywords:** Acute aortic dissection, Oxygenation impairment, Systemic inflammatory response syndrome, C-reactive protein, Albumin

## Abstract

**Background:**

Acute aortic dissection (AAD) is an acute life-threatening cardiovascular disease, which is frequently complicated with oxygenation impairment (OI). We aim to investigate predictors of the development of OI in the patients with AAD.

**Methods:**

We retrospectively collected clinical data of AAD in hypertensive patients from July 2012 to March 2020. The patients included in this study were divided into OI (+) group (oxygenation index≤200) and OI (−) group (oxygenation index> 200). Both groups were compared according to demographic and clinical characteristics, and laboratory findings. Characteristics of hypertension in the patients with AAD were described. Predictors for the development of OI were assessed. And cutoff values were determined by receiver operating characteristics (ROC) curve.

**Results:**

A total of 208 patients were included in this study and the incidence of OI was 32.2%. In OI (+) group, patients had significantly higher peak body temperature (37.85 ± 0.60 vs 37.64 ± 0.44 °C, *P* = .005), higher levels of CRP (42.70 ± 28.27 vs 13.90 ± 18.70 mg/L, *P* = .000) and procalcitonin (1.07 ± 3.92 vs 0.31 ± 0.77μg/L, *P* = .027), and lower levels of albumin (34.21 ± 5.65 vs 37.73 ± 4.70 g/L, *P* = .000). Spearman’s rank correlation test showed that the minimum oxygenation index was positively correlated with albumin, and was negatively correlated with the peak body temperature, serum CRP, procalcitonin, BNP and troponin. The stepwise multiple linear regression analysis showed that the peak body temperature, serum CRP and albumin were independently associated with development of OI. An optimal cutoff value for CRP for predicting OI was ≥9.20 mg/L, with a sensitivity of 91.0% and a specificity of 61.0%.

**Conclusions:**

The peak body temperature, serum CRP and albumin were independent predictors of OI development in the patients with AAD. The serum CRP on admission≥9.20 mg/L might be a valuable and reliable indicator in predicting the development of OI.

## Background

Acute aortic dissection (AAD) is a life-threatening cardiovascular disease with high mortality [1–3]. AAD can cause several organs damage, such as acute lung injury, heart hypofunction, and dysfunction of liver and kidney, etc. Oxygenation impairment (OI) is frequently complicated in the patients with AAD due to acute lung injury, with a reported incidence of up to more than a half [4, 5]. Patients with OI may necessitate mechanical ventilation, and long-term use of ventilator probably can lead to pulmonary infections which can prolong the use of mechanical ventilation and increase intensive-care unit stay. Hence, it is very important to predict OI prior to the occurrence of it in the patients with AAD.

OI remains a great challenge for surgeons, physicians and anesthetists during perioperative periods. Doctors known very little about the pathogenesis, prevention and managing strategy in terms of this severe respiratory complication and some doctors are even not aware of it. Currently the occurrence of OI can’t be predicted at the very early stage of hospitalization, so that the patients with potential occurrence of OI can’t receive appropriate therapeutic management in advance. We consider that interventional therapy before the occurrence of OI was feasible and effective way to achieve better prognosis.

The development of OI is considered to be associated with systemic inflammatory reactions after aortic injury [6, 7]. So the inflammatory makers have been investigated in predicting OI in the patients with AAD in several studies. A recent study indicated that OI in Stanford type-B AAD was correlated with a higher peak level of C-Reactive protein (CRP) and a younger age [6]. Manabu Kurabayashi and his colleagues found that the peak level of CRP and the percentage of the volume of false lumen to aorta were negatively correlated with the occurrence of OI in distal type AAD [8]. Kazunori Tomita et al. reported that body mass index≥22Kg/m^2^, body temperature on admission > 36.5 °C, and oxygenation index on admission < 300 were reliable predictors for OI development [7].

The studies related to the prediction of OI development were all retrospective and observational with relatively small sample size, and conclusions were different. Additionally, chronic inflammation usually exists in the course of hypertension and can be drivers of some cardiovascular diseases [9]. A previous study indicated that early use of beta-blockers could prevent excessive inflammation and OI in distal type AAD [10]. However, it is unknown whether there is a correlation between development of OI and hypertensive characteristics, including hypertension course, hypertension grade, previous blood pressure control, and antihypertensive strategy. For these reasons, it is very necessary to further investigate the predictors of OI development in the patients with AAD. In this present study, we aim to increase the doctors’ awareness of OI development, describe the hypertensive characteristics and investigate the predictors of OI development t in the patients with AAD.

## Methods

### Selection of patients

Our study was conducted in a comprehensive teaching hospital using a retrospective, observational design. The study was approved by the medical ethics committee of our hospital. All data were retrospectively collected from medical records, so written informed consent was not required. And there is no conflict of interest to declare in this study. Between July 2012 and March 2020, a total of 262 consecutive patients were diagnosed with AAD in hypertensive patients. The patients with other etiologies were not included in this present study, such as bicuspid aortic valve disease, Marfan syndrome and trauma, etc. We excluded the patients with elapsed time from the onset of symptoms to admission more than 24 h (*n* = 16), inadequate clinical records (*n* = 1), and patients receiving emergent operations (*n* = 26). We also exclude the patients with concomitant diseases which can impair oxygenation function, including pulmonary infections (*n* = 3), moderate to large pleural effusion (*n* = 2), cardiac dysfunction (*n* = 2), myocardial infarction (*n* = 1), moderate to large hydropericardium (*n* = 2), and acute cerebral infarction (*n* = 1). After applying these exclusive criteria, a total of 208 patients were ultimately included in this present study.

### Study protocol

Data regarding demographic and clinical characteristics, and laboratory findings of the patients were retrospectively collected from their medical records. Hypertensive characteristics were especially collected, including hypertension duration, hypertension grade, antihypertensive therapy, previous blood pressure control and development of hypertensive cardiopathy. Blood samples were obtained on admission and results of laboratory examinations were collected, including blood routing test, CRP, procalcitonin, D-dimer, liver and kidney function, markers of myocardial damage, B-type natriuretic peptide (BNP), and glucose. The arterial oxygen tension (PaO_2_) was measured using an automatic blood gas analyzer system (5700, Instrumentation Laboratory, MA, USA). The baseline value of PaO_2_ was measured at the admission, and we reassessed the value within the first 24 h after admission and at least every 24 h thereafter. The oxygenation index was calculated by PaO_2_ dividing by fraction of inspired oxygen, and OI was defined as minimum oxygenation index≤200. The patients in this study were divided into OI (+) group (oxygenation index≤200) and OI (−) group (oxygenation index> 200). Clinical variables were compared between the two groups, and hypertensive characteristics were described. Correlation between clinical variables and the occurrence of OI were investigated and predictors for OI development were assessed.

All the patients were diagnosed based on the results of contrast medium-enhanced computed tomography. Echocardiography was also performed in the early stage of hospitalization. The type of AAD was classified according to the DeBakey classification.

### Statistical analysis

All statistical analysis was performed using the SPSS 21.0 software for windows (SPSS, Chicago, IL, USA). Mean ± standard deviation, median values, and minimum-maximum values were used for continuous data, while percentages were used for categorical variables. The Student t-test was used for continuous variables between the two groups, and categorical variables were compared using the chi-square test. Spearman’s rank correlation test was used to assess the correlation between the minimum oxygenation index and clinical variables. A stepwise multiple linear regression analysis was performed to determine the independent predictors of OI development. The receiver operating characteristics (ROC) curve analysis was performed to determine the cutoff value of significant clinical variables in prediction of OI. Statistically significant variables (i.e., *P* < .05) found by Spearman’s rank correlation test were included in the stepwise multiple linear regression analysis. Two-tailed *P* value < .05 was considered to be statistically significant.

## Results

**Fig. 1:**
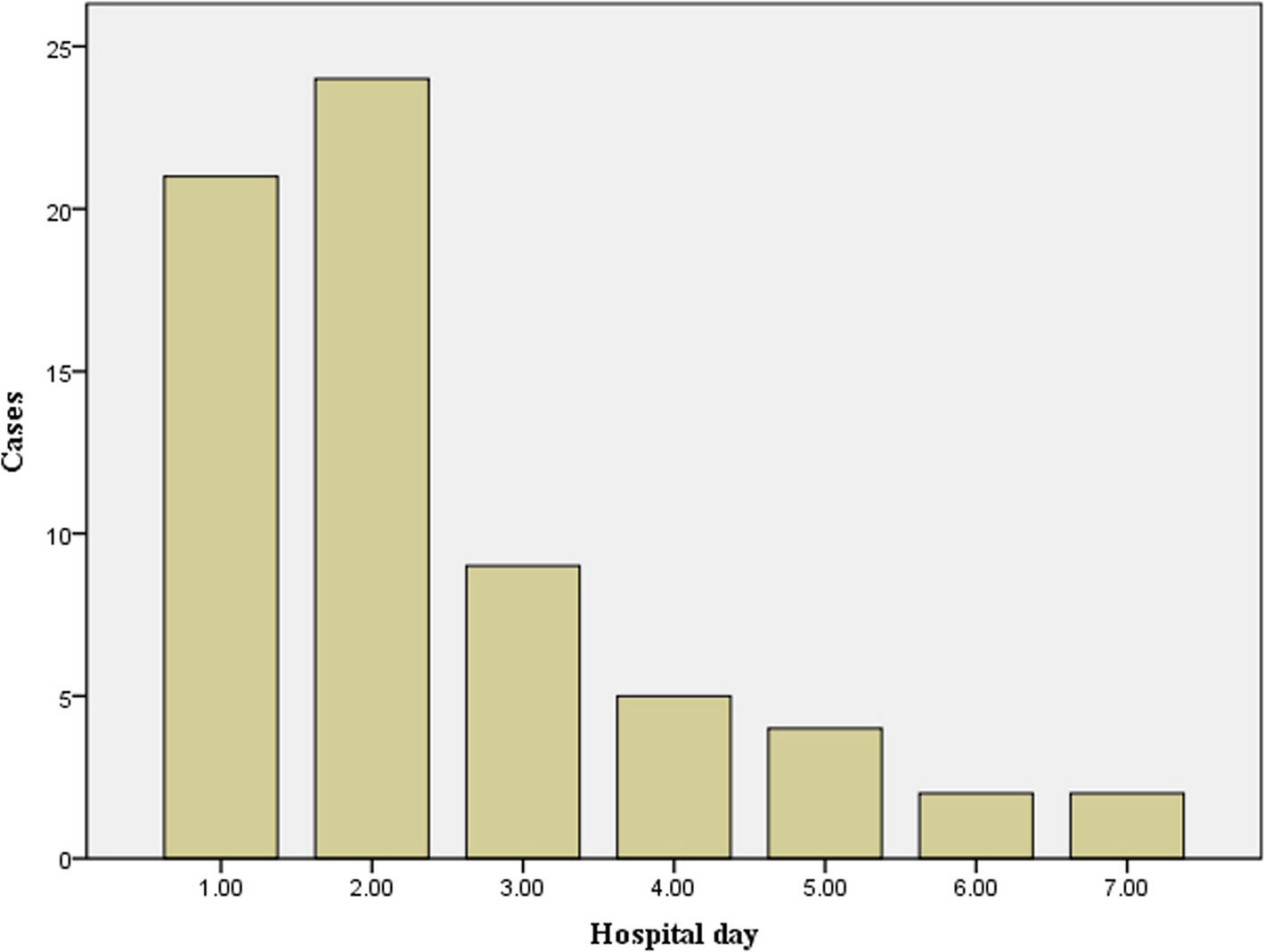
The timing of occurrence of oxygenation impairment after hospitalization

The baseline demographic and clinical characteristics of the patients are summarized in Table 1. The mean age was 56.6 ± 12.6 years, ranging from 26 to 87 years; 171 (82.2%) patients were male. The development of OI was observed in 67 (32.2%) patients on hospital day 2.4 ± 1.5 on average (Fig. 1). The elapsed time from onset of symptoms to admission to hospital was 9.90 ± 6.92 h, ranging from 1.0–24.0 h, showing no significant difference between the two groups. Mechanical ventilation was used in 22 (32.8%) patients in OI (+) group, including 18 (26.9%) patients with invasive mechanical ventilator and 4 (6.0%) patients with non-invasive mechanical ventilator. All the patients were diagnosed with hypertension, and hypertensive characteristics were shown in Table 2. 153 (73.56%) patients had a history of hypertension with 8.0 ± 6.7 years (ranging from 3 months-40 years), and the other 55 (26.44%) patients were diagnosed with hypertension after admission to hospital.

**Table 1:** Baseline demographic characteristics, clinical features, and laboratory results of the patients ^a^

Variables	OI (+) group (*n* = 67)	OI (−) group (*n* = 141)	*P*
Age (years)	56.8 ± 12.6	56.6 ± 12.6	0.896
Gender (male/female)	51/16	120/21	0.124
Body mass index	25.37 ± 4.68	25.29 ± 2.99	0.869
Elapsed time after onset (hours)	10.7 ± 6.7	9.5 ± 7.0	0.263
Current smoker	56.72% (38)	59.57% (84)	0.764
Alcohol drinking	44.78% (30)	41.13% (58)	0.654
Body temperature (admission) (°C)	36.59 ± 0.27	36.55 ± 0.28	0.436
The peak body temperature (°C)	37.85 ± 0.60	37.64 ± 0.44	0.005
Heart rate (beats per minute)	84.4 ± 16.5	82.1 ± 15.7	0.331
Respiratory rate (breaths per minute)	20.1 ± 2.4	19.9 ± 3.0	0.584
Systolic blood pressure (mmHg)	155.8 ± 34.1	156.2 ± 28.6	0.925
Diastolic blood pressure (mmHg)	90.2 ± 19.5	90.2 ± 19.4	0.999
Platelet count (× 10^9^/L)	165.8 ± 60.5	164.7 ± 68.4	0.900
White blood cell count (×10^9^/L)	12.31 ± 3.38	12.50 ± 3.91	0.745
Neutrophil percentage (%)	86.19 ± 7.18	85.36 ± 7.25	0.245
C-Reactive protein (mg/L)	42.70 ± 28.27	13.90 ± 18.70	0.000
Procalcitonin (ug/L)	1.07 ± 3.92	0.31 ± 0.77	0.027
D-dimer (mg/L)	9.51 ± 12.28	9.30 ± 13.43	0.912
Albumin (g/L)	34.21 ± 5.65	37.73 ± 4.70	0.000
Serum creatinine (umol/L)	102.13 ± 78.44	91.13 ± 44.67	0.200
Creatine kinase-muscle/brain (ug/L)	2.88 ± 5.37	3.55 ± 10.09	0.608
Troponin (ug/L)	0.029 ± 0.058	0.033 ± 0.079	0.654
B-type natriuretic peptide (ng/L)	124.76 ± 73.00	150.81 ± 183.48	0.264
Serum glucose (mmol/L)	6.90 ± 2.27	6.98 ± 2.36	0.812
DeBakey classification			0.749
Type I	29.85% (20)	31.91% (45)	
Type II	0	1.42% (2)	
Type IIIa	4.48% (3)	4.26% (6)	
Type IIIb	65.67% (44)	62.41% (88)	

^a^ *P* < .05 was considered as significant

**Table 2:** Characteristics of hypertension in the patients with acute aortic disease ^a^

Variables	OI (+) group (*n* = 67)	OI (−) group (*n* = 141)	*P*
Hypertension duration	5.87 ± 6.89	5.99 ± 6.75	0.909
Hypertension grade			0.437
Grade 1	8.96% (6)	9.22% (13)	
Grade 2	20.90% (14)	29.08% (41)	
Grade 3	70.15% (47)	61.70% (87)	
Antihypertensive agent			0.109
No antihypertensive therapy	73.13% (49)	75.89% (107)	
Angiotensin-converting enzyme inhibitors	11.11% (2/18)	11.76% (4/34)	
Angiotensin receptor blockers	33.33% (6/18)	26.47% (9/34)	
Beta-blockers	0	5.88% (2/34)	
Calcium channel blockers	61.11% (11/18)	58.82% (20/34)	
Chinese traditional medicine	11.11% (2/18)	11.76% (4/34)	
Combination therapy/ Monotherapy	3/18	5/34	1.000
Previous blood pressure control			0.566
< 120 mmHg	0	0.71% (1)	
120-140 mmHg	5.97% (4)	9.22% (13)	
> 140 mmHg	94.03% (63)	90.07% (127)	
Hypertensive cardiopathy	52.24% (35)	60.28% (85)	0.295

^a^ *P* < .05 was considered as significant

Compared with OI (−) group, patients in OI (+) group had significantly higher peak body temperature, higher levels of CRP and procalcitonin, and lower levels of albumin (Table 1). The body temperature peaked on 3.1 ± 1.4 days in OI (+) group and 2.9 ± 1.3 days in OI (−) group on average. No significant differences were indicated by statistical analysis in the terms of other inflammation related markers, including platelet count, white blood cell count and neutrophil percentages. Distribution of the patients’ DeBakey classification and hypertensive characteristics showed similar between the two groups.

**Fig. 2:**
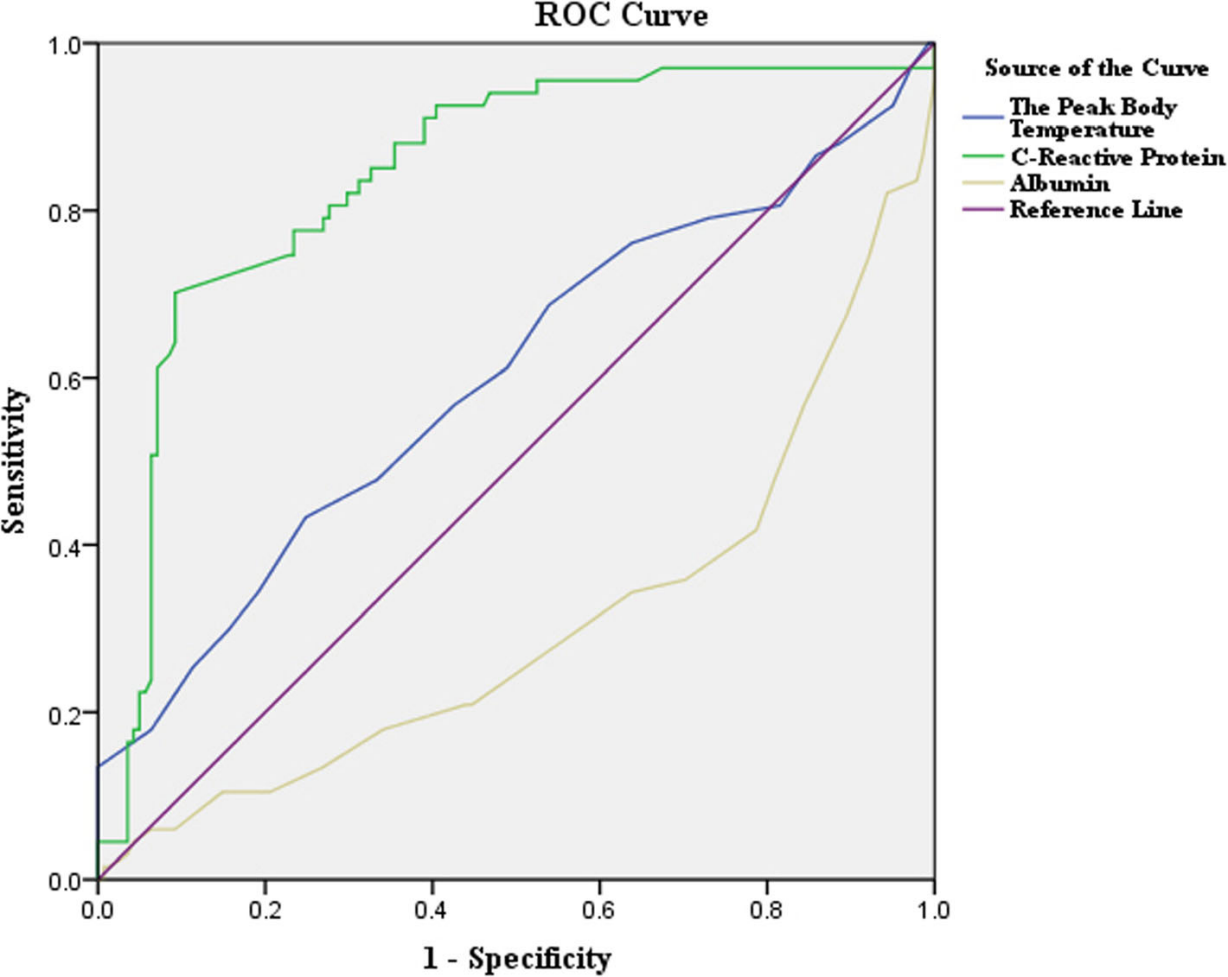
Receiver operating characteristic (ROC) curves of C-reactive protein (CRP), albumin and peak body temperature for the development of oxygenation impairment in the patients with acute aortic dissection. The area under curve (AUC) for CRP was 0.840 (95% confidence interval [CI]: 0.780–0.901). The AUC for albumin was 0.306 (95% CI: 0.225–0.387). The AUC for peak body temperature was 0.600 (95% CI: 0.514–0.687)

Table 3 showed that the minimum oxygenation index was significant and positively correlated with albumin, and was negatively correlated with the peak body temperature, serum CRP, procalcitonin, troponin and BNP in Spearman’s rank correlation test. Correlation between hypertensive characteristics and development of OI was not indicated by statistical analysis. The other variables with weak associations were subsequently excluded for stepwise multiple linear regression analysis. Serum CRP, albumin and peak body temperatures were found to be independent predictors of the occurrence of OI in stepwise multiple linear regression analysis (Table 4). In ROC curve analysis, an optimal cutoff value for CRP in predicting OI was ≥9.20 mg/L, with a sensitivity of 91.0% and a specificity of 61.0%. The sensitivity and specificity of CRP were indicated to be reliable in the prediction of OI development due to significant larger area under curve (AUC) than peak body temperature and albumin (Fig. 2). The AUC for CRP was 0.840 (95% CI: 0.780–0.901). The AUC for albumin was 0.306 (95% CI: 0.225–0.387), and the AUC for peak body temperature was 0.600 (95% CI: 0.514–0.687).

**Table 3:** Correlation between clinical variables and minimum oxygenation index indicated by the Spearman rank correlation test^a^

Variables	*r*	*P*
Age	−0.001	0.987
Male	− 0.094	0.176
Body mass index	−0.067	0.339
Elapsed time after onset	−0.039	0.580
Current smoker	−0.011	0.871
Alcohol drinking	−0.090	0.194
Hypertension duration	−0.011	0.870
Hypertension grade		
Grade 1	0.039	0.573
Grade 2	0.015	0.830
Grade 3	−0.022	0.755
Antihypertensive agent		
No antihypertensive agent	0.019	0.787
Angiotensin-converting enzyme inhibitors/Angiotensin receptor blockers	−0.027	0.700
Beta-blockers	−0.027	0.702
Calcium channel blockers	−0.013	0.854
Chinese traditional medicine	−0.032	0.651
Combination therapy of antihypertensive agents	−0.064	0.356
Previous blood pressure control		
< 120 mmHg	−0.092	0.185
120-140 mmHg	0.069	0.321
> 140 mmHg	0.101	0.145
Hypertensive cardiopathy	−0.019	0.781
Body temperature on admission	0.005	0.942
The peak body temperature	−0.190	0.006
The timing of the peak body temperature	−0.101	0.148
Heart rate	−0.053	0.448
Respiratory rate	−0.040	0.568
Systolic blood pressure	0.064	0.362
Diastolic blood pressure	0.022	0.747
Platelet count	0.019	0.788
White blood cell count	−0.023	0.742
Neutrophil percentage	−0.033	0.637
C-Reactive protein	−0.420	0.000
Procalcitonin	−0.149	0.032
D-dimer	−0.101	0.147
Albumin	0.271	0.000
Serum creatinine	−0.094	0.176
Creatine kinase-muscle/brain	−0.086	0.218
Troponin	−0.170	0.014
B-type natriuretic peptide	−0.167	0.016
Serum glucose	0.054	0.434
DeBakey classification	0.051	0.466

^a^ *P* < .05 was considered as significant

**Table 4:** A stepwise multiple linear regression analysis of the clinical variables associated with the minimum oxygenation index ^a^

Minimum oxygenation index	Coefficient	Standard error	*t*	*P*
C-Reactive protein	−1.582	0.272	−5.816	0.000
Albumin	4.797	1.340	3.579	0.000
Peak body temperature	−42.629	13.873	−3.073	0.002

^a^ *P* < .05 was considered as significant

## Discussion

In our study, OI occurred in 67 patients (32.2%) on hospital day 2.4 ± 1.5 on average, and 22 patients (32.8%) in OI group necessitated mechanical ventilation. The high incidence of OI development in AAD patients and risks of use of mechanical ventilation highlight the importance to predict this severe respiratory complication. However, limited is known about the pathogenesis, prevention and management. This study indicated that the occurrence of OI can be predictable in the patients with AAD. Elevated serum CRP levels and peak body temperature, and decreased albumin levels were independent predictor of OI development.

Inflammatory cells and cytokines have been found in participating in the pathogenesis of aortic dissection [11, 12]. The first stage of inflammatory reactions is the long-term chronic inflammation in the aorta in the patients with hypertension. Inflammation, oxidative stress and genetic predisposition play great roles in the pathogenesis of hypertension [13]. Chronic low-grade inflammation exists in the course of hypertension, triggers the progress of hypertension and contributes to vascular remodeling [9, 14, 15]. Angiotensin II also serves an important inflammatory roles in hypertension; it can induce macrophages to differentiate into the M-1 type, secrete proinflammatory cytokines, and stimulate the attachment and migration of leukocytes [15, 16]. The second stage of inflammatory reactions is the rapid development to acute inflammation after aortic injuries [5, 6]. And it can subsequently develop to systemic inflammatory reactions syndrome (SIRS) due to uncontrolled inflammatory cascade [17]. Aortic samples from the patients with aortic dissection revealed a large amount of macrophages infiltration [18]. A large number of cytokines have also been found infiltrating the aortic wall of patients with aortic dissection, such as IL-6 and IL-17 [12]. It was reported that levels of IL-11 and interferon γ were increased in blood samples in acute thoracic aortic dissection [19]. In this present study, there was no correlation between the development of OI and hypertension grade, hypertension course, previous blood control, and antihypertensive strategy, suggesting that the impact of chronic inflammation in hypertension is limited in the development of OI. It was majorly due to the second stage of inflammatory reactions and the severity of SIRS.

Systemic inflammatory reactions play key roles in the pathogenesis of acute lung injuries and the development of OI. Recent studies found that AAD complicated with acute lung injury was highly associated with the macrophages infiltrating the pulmonary interstitial tissue and released matrix metalloproteinases 9 in response to angiotensin II [20, 21]. Inflammatory reactions can cause fluid to leak across alveolar-capillary barrier because of increased permeability and produce enough alveolar edema to cause the clinical characteristics of refractory hypoxemia [22]. In mice model, IL-22 could significantly attenuate the incidence and severity of angiotensin II induced acute lung injury [23]. Hence, concerns on anti-inflammatory agents and some protective cytokines will help find insights on the therapeutic strategy for acute lung injury and OI development in AAD. Very limited has been known about the pathogenesis of acute lung injury and OI development in the patients with AAD, so further studies need to be conducted.

Given the great roles of inflammatory reactions in the development of OI, inflammatory markers should be especially investigated in predicting OI development in the patients with AAD. Inflammatory markers can reflect the severity of SIRS, and in turn it may be predictors of OI development. CRP, a highly sensitive marker of systemic inflammation, is an acute-phase protein induced by pro-inflammatory cytokines, particularly IL-6 [24]. Elevated CRP was indicated in the patients with AAD [25], and it can be a prognostic factor to be independently associated with inhospital death [24, 26]. Procalcitonin has also been advocated as a diagnostic parameter in systemic inflammation and infectious diseases [27]. Recent studied showed that postoperative levels of procalcitonin could be used to predict adverse outcome in acute type-A aortic dissection [28, 29]. We first described the characteristics of procalcitonin in the patients with OI in AAD. Our study showed the negative correlation between the 2 inflammatory markers, CRP and procalcitonin, and the development of OI. The peak body temperature was significantly elevated in the patients with OI and negatively correlated with the occurrence of OI, further increasing the evidence that systemic inflammatory reactions contribute to the development of OI in the patients with AAD. Upon an inflammatory challenge, hepatic and pulmonary macrophages (and later brain endothelial cells) start to release lipid mediators, including prostaglandin E_2_ and cytokines [30]. Blood prostaglandin E_2_ enters the brain and triggers fever, and at later stages of fever, prostaglandin E_2_ synthesized within the blood-brain barrier maintains fever [30].

Albumin is both an important nutritional indicator and an acute-phase protein involved in inflammatory response [31]. Lower serum albumin levels were indicated in SIRS [32]. Decreased albumin levels have also been found in some cardiovascular diseases, of which inflammatory reaction participates in the pathogenesis, including heart failure and coronary heart disease [33, 34]. Its synthesis is stimulated by hormones and can be inhibited by pro-inflammatory substances, including IL-6 [35]. In inflammatory states, albumin distribution alters from intravascular to extravascular compartments due to increased microvascular permeability [36, 37]. And increased depletion of albumin further leads to the negative albumin balance [37]. We firstly introduce the value of albumin in predicting OI in the patients with AAD. We guess that decreased levels of albumin in the patients with OI are perhaps due to more severe inflammatory reactions.

Stepwise multiple linear regression analysis showed that elevated serum CRP levels, higher peak body temperature and decreased albumin levels were independent predictors of the occurrence of OI. However, the average timing of peak body temperature was later than that of the occurrence of OI, and only CRP had a good sensitivity and specificity indicated by ROC curves. Hence, the predicting value of the peak body temperature and albumin was actually limited. Our study showed that the serum CRP ≥ 9.20 mg/L might be a valuable predictor of OI development in AAD patients. CRP is a simple, widely available, and in-expensive test. So it can be widely used as a biomarker in predicting OI in the patients with AAD.

This study also found that most of AAD patients had poor blood pressure control at home. Most of them were grade 3 hypertension and nearly a half had hypertensive cardiopathy. Patients had poor awareness of blood pressure monitoring and regular antihypertensive therapy. Therefore, it highlights the importance of the education of hypertension in public, especially blood pressure monitoring and regular antihypertensive therapy in patients with hypertension.

This study has some limitations. Our study is a single-center retrospective study, and the sample size is still not large enough. We will further investigate the expression differences of some cytokines including IL-6 in future. We hope to establish a multicenter, large-sample prospective cohort study focusing on the prevention and management of OI in AAD patients.

## Conclusions

The serum CRP on admission≥9.20 mg/L may be a valuable inflammatory marker in predicting the development of OI in the patients of AAD. And higher peak body temperature and decreased albumin levels may also be helpful indicators of the occurrence of OI. These findings will help identify the patients who are susceptible to have high risks of developing OI, thereby leading to prevention and appropriate interventional therapies prior to the occurrence of OI to gain better prognosis.

## Data Availability

The datasets used and/or analyzed in this present study are available from the corresponding author on reasonable request.

## Abbreviations

AAD: Acute aortic dissection
OI: Oxygenation impairment
CRP: C-Reactive protein
BNP: B-type natriuretic peptide
PaO2: Arterial oxygen tension
ROC: Receiver operating characteristics
AUC: Area under curve
SIRS: Systemic inflammatory response syndrome
